# A multiscale tissue assessment in a rat model of mild traumatic brain injury

**DOI:** 10.1093/jnen/nlac100

**Published:** 2022-11-04

**Authors:** Isabel San Martín Molina, Michela Fratini, Gaetano Campi, Manfred Burghammer, Tilman A Grünewald, Raimo A Salo, Omar Narvaez, Manisha Aggarwal, Jussi Tohka, Alejandra Sierra

**Affiliations:** A.I. Virtanen Institute for Molecular Sciences, University of Eastern Finland, Kuopio, Finland; Institute of Nanotechnology-CNR c/o Physics Department, Sapienza University of Rome, Rome, Italy; IRCCS Fondazione Santa Lucia, Rome, Italy; Institute of Crystallography, CNR, Rome, Italy; European Synchrotron Radiation Facility, Grenoble Cedex, France; European Synchrotron Radiation Facility, Grenoble Cedex, France; Aix-Marseille Université, CNRS, Centrale Marseille, Institut Fresnel, Marseille, France; A.I. Virtanen Institute for Molecular Sciences, University of Eastern Finland, Kuopio, Finland; A.I. Virtanen Institute for Molecular Sciences, University of Eastern Finland, Kuopio, Finland; Russell H. Morgan Department of Radiology and Radiological Science, John Hopkins University School of Medicine, Baltimore, Maryland, USA; A.I. Virtanen Institute for Molecular Sciences, University of Eastern Finland, Kuopio, Finland; A.I. Virtanen Institute for Molecular Sciences, University of Eastern Finland, Kuopio, Finland

**Keywords:** Axonal damage, Cell counting, Diffusion tensor imaging, Mild traumatic brain injury, Myelinated axons, Scanning micro-X-ray diffraction, Structure tensor analysis

## Abstract

Diffusion tensor imaging (DTI) has demonstrated the potential to assess the pathophysiology of mild traumatic brain injury (mTBI) but correlations of DTI findings and pathological changes in mTBI are unclear. We evaluated the potential of ex vivo DTI to detect tissue damage in a mild mTBI rat model by exploiting multiscale imaging methods, histology and scanning micro-X-ray diffraction (SμXRD) 35 days after sham-operation (n = 2) or mTBI (n = 3). There were changes in DTI parameters rostral to the injury site. When examined by histology and SμXRD, there was evidence of axonal damage, reduced myelin density, gliosis, and ultrastructural alterations in myelin that were ongoing at the experimental time point of 35 days postinjury. We assessed the relationship between the 3 imaging modalities by multiple linear regression analysis. In this analysis, DTI and histological parameters were moderately related, whereas SμXRD parameters correlated weakly with DTI and histology. These findings suggest that while DTI appears to distinguish tissue changes at the microstructural level related to the loss of myelinated axons and gliosis, its ability to visualize alterations in myelin ultrastructure is limited. The use of several imaging techniques represents a novel approach to reveal tissue damage and provides new insights into mTBI detection.

## INTRODUCTION

Mild traumatic brain injury (mTBI) is a major public health problem worldwide affecting approximately 200–700 out of 100 000 people per year (1, 2). Clinically, mTBI is defined by a loss of consciousness for 30 minutes or less, confusion or disorientation, and/or amnesia for less than 24 hours (3, 4). However, even after a mild injury, many patients suffer long-term consequences, such as depression, sleep, or cognitive problems, as well as a predisposition to develop neurodegenerative diseases, such as Alzheimer or Parkinson disease (5, 6). At the cellular level, the primary injury triggers a complex cascade of events that can compromise cerebral function (7–9). The common hallmarks of this kind of injury are diffuse axonal injury, inflammation, neurodegeneration, or vasculature shearing, occurring at the level of the primary injury, as well as spreading to areas in the so-called the secondary injury (10–12). However, clarifying the relationship between clinical outcomes of mTBI patients and the underlying tissue pathology remains a challenge (13).

Noninvasive imaging methods, such as computed tomography (CT) or magnetic resonance imaging (MRI), are the gold standard techniques used in the diagnosis of TBI patients in the clinic (14, 15). While CT is commonly used to assess skull fractures or parenchymal bleeding (16, 17), MRI, such as T2- and T2* weighted imaging, is useful for assessing brain atrophy, edema, hemorrhages or microbleeds (18, 19). These techniques effectively detect major structural abnormalities in moderate and severe cases of TBI but they have a more limited ability to detect subtle tissue alterations occurring after a mild injury (20). Techniques based on diffusion MRI (dMRI), eg diffusion tensor imaging (DTI), have been used to detect tissue microstructural damage after mTBI in both clinical (21–23), and experimental studies (24–26). Experimental mTBI studies have revealed changes in DTI parameters associated with inflammatory processes, reduced myelin density and/or axonal damage, in both white and grey matter areas (27–33). Despite the potential of DTI, it remains to be demonstrated which processes are associated with the changes occurring in DTI parameters as well as its limits to detect minor changes after a mild injury.

In this study, we used a multiscale imaging approach to evaluate the potential for DTI to detect the tissue damage in the rat brain at 35 days after mTBI. We used ex vivo DTI to assess tissue microstructural changes, light microscopy to examine disturbances in the cellularity and morphology of myelinated axons and scanning micro-X-ray diffraction (SμXRD) to evaluate the ultrastructure of myelin at the nanometer scale. Subsequently, we devised a regression model to examine the relationships between these 3 techniques in terms of tissue structure. This study highlights the potential pros and cons of DTI for detecting tissue microstructural changes after mTBI by using a multiscale imaging approach.

## MATERIALS AND METHODS

### Animals and mTBI model

Adult male Sprague-Dawley rats were used in all the experiments (10 weeks old, 300–350 g, Harlan Netherlands B.V, Venray, Netherlands). They were housed individually in cages in a climate-control room under a 12-hour/12-hour light/dark cycle with ad libitum diet. All animal procedures were approved by the Animal Ethics Committee of the Province Government of Southern Finland and carried out according to the guidelines set by the European Community Council Directives 2010/63/EEC.

Detailed experimental procedures were performed as in (32). In brief, the rats were anesthetized with an i.p. injection (6 ml/kg) containing a mixture of sodium pentobarbital (58 mg/kg), chloral hydrate (60 mg/kg), magnesium sulfate (172 mg/kg), propylene glycol (42.8%), and absolute ethanol (11.6%). A craniotomy (∅ = 5 mm) was made between bregma and lambda on the left convexity (anterior edge 2 mm posterior to bregma, lateral edge adjacent to the left lateral ridge). At this point, sham-operated animals (n = 2) were not subjected to the impact and were kept in the recovery room. For the mTBI animal group, after the craniotomy, we used the lateral fluid percussion injury model to induce a mild TBI (n = 3), in which a fluid percussion device (AmScien Instruments, Richmond, VA) was utilized to produce a transient fluid pressure (21–23 ms) against the exposed dura. We used an impact pressure of 0.97 ± 0.06 atm to induce a mild brain injury in the 3 rats. After the surgical procedures, we monitored the animals by assessing the right-time reflex latency, apnea time, seizure post-mTBI and hematoma.

### Ex vivo DTI acquisition and data processing

At 35 days post-mTBI, all of the rats were anesthetized with 5% isoflurane in 70% nitrogen/30% oxygen, after which they were transcardially perfused with 0.9% NaCl for 5 minutes (30 mL/minute) followed by 4% paraformaldehyde in 0.1 M PB pH 7.4 for 25 minutes (30 mL/minute). After perfusion, the brains were removed from the skull and postfixed in 4% PFA for 4 hours. Before ex vivo DTI evaluation, the brains were transferred to a solution containing 1 mM gadopentetate dimeglumine (Magnevist, Berlex Imaging, Wayne, NJ) in 0.1 M PBS for at least 72 hours. In order to achieve an effective suppressed background signal imaging acquisition, we placed the brains inside a polyethylene tube filled with perfluoro-polyether (Fomblin, Solvay, Inc., Princeton, NJ).

The fixed rat brains were scanned in an 11.7 T NMR spectrometer (Bruker BioSpin, Billerica, MA) with a Micro2.5 gradient system (maximum gradient strength = 1000 mT/m) using a birdcage volume coil (diameter = 20 mm) for transmission and signal reception. A 3D diffusion-weighted gradient- and spin-echo (DW-GRASE) sequence (34) was used with the following parameters: TE = 33 ms, TR =800 ms, rare-factor/EPI factor = 4/3, number of averages = 2, bandwidth = 100 kHz, matrix size = 152 × 112 × 78, FOV = 22.8 × 16.8 × 11.7 mm^3^, number of b0 images = 4, 30 directions per b-value (Δ  =  12 ms, δ  =  5 ms, b-value = 3000 and 6000 s/mm^2^), resolution of 0.15 mm^3^ isotropic (zero-filling interpolation to 0.075 mm^3^ isotropic) scan time of ∼21 hours.

For the ex vivo DTI analyses, the k-space data using an in-house code in IDL (ITT Visual Information Solutions, Boulder, CO) was first processed to reconstruct the DW data into Nifti format. The reconstructed DW data were then preprocessed using MRtrix3 software (35) by performing image denoising based on random matrix theory (36) and then Gibbs’ ringing removal using the local subvoxel-shifts method (37). Then, a bias field correction was applied to remove spatial intensity inhomogeneities (38); motion and eddy current correction was performed using Advanced Normalization Tools software (39). All DTI maps were then created as previously described (33).

### Histological staining and quantitative analyses

After ex vivo DTI imaging, the brains were washed in 0.9% NaCl for at least 2 hours at 4°C and then, cryoprotected for 36 hours in a cryoprotective solution (20% glycerol in 0.02 M KPBS, pH 7.4). After cryoprotection, the brains were frozen in dry ice and stored at −70°C until cutting. Brains were sectioned in the coronal plane with a sliding microtome (30 µm, 1-in-5-series). The first series of sections were stored in 10% formalin, and the 4 remaining series in cryoprotectant-tissue collecting solution (30% ethylene glycol, 25% glycerol in 0.05 M sodium phosphate buffer) at −20°C until processing.

Nissl (thionin) staining of the first series of the sections were performed to assess the cytoarchitectonics, gliosis or increased cell density (CD), and the extent of neurodegeneration at 35 days post-mTBI. To assess cell density, high-resolution photomicrographs of the whole brain section (tiles, 0.013 μm^2^/pixel) were acquired using a ZeissAxioImager2 light microscope equipped with a digital camera (Zeiss Axiocam color 506). Each photomicrograph of Nissl-stained sections was analyzed using an in-house developed MATLAB code for automated cell counting analysis available at https://github.com/aAbdz/cell_counter.

For the second series of sections, gold chloride was used to assess axonal and myelin damage (40). When deriving anisotropy from each image, we used the structure tensor (ST)-based method (41) on high-resolution photomicrographs of myelin-stained sections (0.013 μm^2^/pixel). We calculated the anisotropy index (AI) as a histological derived parameter by applying the pixelwise ST-based method (32).

### SμXRD acquisition and data analysis

One section per animal at −2.00 mm from bregma was selected from the third series of brain sections, rostrally to the lesion site (−3.60 mm from bregma). SμXRD measurements were performed on the ID13 beamline of the European Synchrotron Radiation Facility ([ESRF], Grenoble, France). Each brain section was mounted on a 4-µm-thin ultralene film, which was glued to a ring holder. The X-ray beam was monochromatized with a liquid nitrogen cooled Si-111 double monochromator and then focused by compound refractive lenses on a 2 × 2 μm^2^ spot with an energy of 12.6 keV. The samples were scanned by a step motor stage with 0.5 µm repeatability, using a step size of 2 μm in both the vertical, z, and the horizontal, y. The exposure time selected for SµXRD measurements was tested beforehand. We did not observe apparent changes in 30 XRD patterns continuously collected in the same spot (2 µm^2^ × 2 µm^2^). Therefore, an exposure time of about 10 ms was used for each frame in each 2 µm^2^ × 2 µm^2^ spot, avoiding in this way the radiation damage in our SµXRD measurements and at the same time, ensuring sufficient photon counts statistics. At each point reached by the y–z translator, we collected a 2D diffraction frame in transmission geometry using a fast Eiger 4 M detector (Dectris) 135 mm distant from the sample. The beam center, detector tilt and sample-to detector distance were calibrated with a NIST SRM 676 Al_2_O_3_ standard using pyFAI.

**Figure 1.**
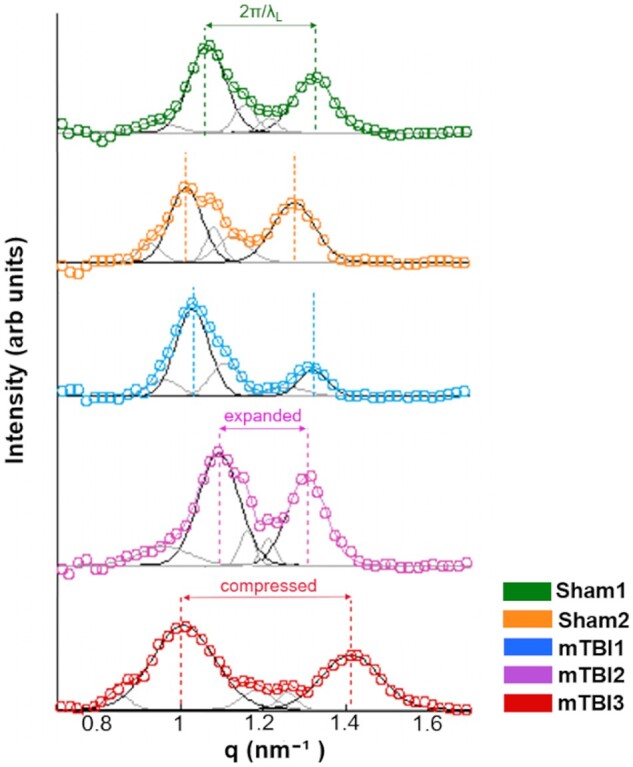
X-ray diffraction profiles after normalization with respect to the incident X-ray flux. Then, all spectra were subtracted from the background, that is, XRD profiles from areas without myelin content as background profiles. The thick colored lines along the experimental data (circles) result from the fitting procedure using the Gaussian line shape. The black and grey Gaussian peaks correspond to the Bragg peaks of the multilamellar and hexagonal phases, respectively. arb. units, arbitrary units.

For SμXRD image processing, we radially integrated the 2D diffraction patterns. The data were then normalized with respect to the incident flux of the X-ray beam to account for temporal variations and subtracted the background (the XRD profile measured in areas without myelin peaks) by obtaining the 1D intensity profiles, I, versus momentum transfer

$$q=\;\frac{4\pi \sin\theta }{\lambda },$$

where λ is the X-ray incident wavelength and θ is the scattering angle. The small beam size made it possible to use a beam stopper small enough to work with a short sample-detector distance. In this way, we achieved the simultaneous acquisition of Synchrotron wide-angle X-ray scattering (WAXS) and small-angle X-ray scattering (SAXS) patterns for each investigated point, covering a q-range of [0.1–12] nm^−1^ (42). In the SAXS regime, we distinguished between 2 intensity components I(q) = I_L_(q) + I_H_(q) ascribed to a main multilamellar phase (L) and a minority hexagonal phase (H), respectively (43). As shown in Figure 1, the I_L_(q) contribution presents the quasi-Bragg peaks at:

$$q_{L}\left(h\right)=\frac{2\pi h}{\lambda_{L}}$$

where *h* = 2, 3, and λ_L_ is the mean interlamellar separation representing the myelin period or lamellar lattice unit (44). Beyond these main peaks, we obtained three peaks corresponding to the planar hexagonal minority phase, H. These peaks have been indexed by

$$q_{H}\left(h,\;k\right)=\frac{4\pi }{\sqrt{3}\lambda_{H}}\sqrt{h^{2}+\mathrm{hk}+k^{2}},$$

where (h, k) are (1, 0), (2, 0), and (2, 3) reflections (with corresponding multiplicity) and λ_H_ represents the hexagonal lattice unit. All the I(q) profiles were fitted by Gaussian line shapes to provide the lattice units λ_L_ and λ_H_ in the lamellar and hexagonal phases and the myelin content (C) in lamellar (C_L_) and hexagonal (C_H_) phases by (45):

$$C_{\mathrm{Phase}}=\int I_{\mathrm{Phase}}\left(q\right)dq,$$

where I_Phase_ is I_L_(q) or I_H_(q) for the lamellar and hexagonal phase, respectively. The period and the content of the lamellar and hexagonal phase in each selected region of interest (ROI) were mapped. We defined a commensurability parameter, η, given by the ratio between the unit cell of the lamellar and hexagonal phases (η = λ_L_/λ_H_). Image processing and data analyses were performed by using a customized in-house developed code written in MATLAB (R2012b; MathWorks, Natick, MA) (46).

### ROI analyses of DTI, histology, and SμXRD

An ROI-based approach on specific selected brain areas was used to extract imaging parameters with each technique. We manually outlined ROIs in the external capsule, layer VI of the somatosensory cortex, cingulum, corpus callosum and internal capsule at approximately −2.00 mm from bregma ipsilaterally to the sham-operation or injury in the brain. The location of the ROIs in the 3 methodologies was anatomically delineated in the same location by an expert (I.S.M.M.) (Supplementary Fig. S1), and ensured the same anatomical location in the 3 imaging techniques using anatomical landmarks.

An in-house software (AEDES, http://aedes.uef.fi/) in MATLAB (R2020b; MathWorks) was used to derive the ROI values from the ex vivo DTI parameters. The ZEN software (version 3.1; Carl Zeiss Microscopy, Jena, Germany) was used in the evaluation of the high-resolution photomicrographs of Nissl and myelin staining. For SμXRD, the ROIs using an in-house MATLAB code in the intensity maps were outlined. GraphPad Prism (version 5.03, GraphPad Software Inc. La Jolla, CA), KaleidaGraph (version 3.6, Synergy Software, Reading, PA) and MATLAB (R2012b; MathWorks) software were used to obtain the graphs.

### Statistical analyses

We qualitatively described the difference between sham-operated and mTBI animals due to low sample size. For DTI results, we denoted values from mTBI animals as higher (lower) when the values from all the 3 mTBI animals were higher (lower) than the values from the values from the 2 sham-operated animals. Similarly, as in DTI, for the histological findings, we reported changes from a single mTBI animal when the histological metrics values were higher (lower) than the values from both sham-operated animal values. For SµXRD results, the measured values in each individual animal were compared to the standard values for the lamellar period and commensurability of myelin reported in the literature (47, 48).

The relationship between DTI, histology and SμXRD was assessed by constructing a multiple linear regression model in which parameters from one imaging modality were used to explain parameters from a second modality (49). Therefore, we constructed models where DTI parameters were used to explain histology parameters; SμXRD parameters were used to explain DTI parameters, etc. We used the following multiple linear regression model

$$y_{kj}=b^{T}x_{kj}+c+e_{kj}$$

where $y_{kj}$ is the parameter to be explained and ***x***_kj_ is the vector of predictor parameters for the region *j* of the animal k. ***b*** and *c* are the regression parameters, and *e*_kj_ represents normally distributed i.i.d. errors. The parameter to be explained and predictor parameters originate from different modalities. For DTI, the following were considered: fractional anisotropy index (FA), axial diffusivity (AD), radial diffusivity (RD), mean diffusivity (MD), linear anisotropy index (CL), planar anisotropy index (CP), and spherical anisotropy index (CS); for histology, anisotropy index (AI) and cell density (CD) were selected; for diffraction, lamellar phase content (C_L_), lamellar phase period (λ_L_), hexagonal phase period (λ_H_), and hexagonal phase content (C_H_) were selected. The regions evaluated were external capsule, layer VI of the somatosensory cortex, cingulum, corpus callosum and internal capsule (Supplementary Fig. S1). It should be noted that when DTI was used to explain other modalities, the regression model excluded RD and CL parameters due to structural collinearity, indicative of correlations between two or more predictor variables. When fitting the regression model and estimating the 95% CI values for *R*^2^, we used the SPSS code from (50) in SPSS (version 27, IBM SPSS Statistics, Chicago, IL).

## RESULTS

### Alterations in ex vivo DTI parameters after mTBI

**Figure 2.**
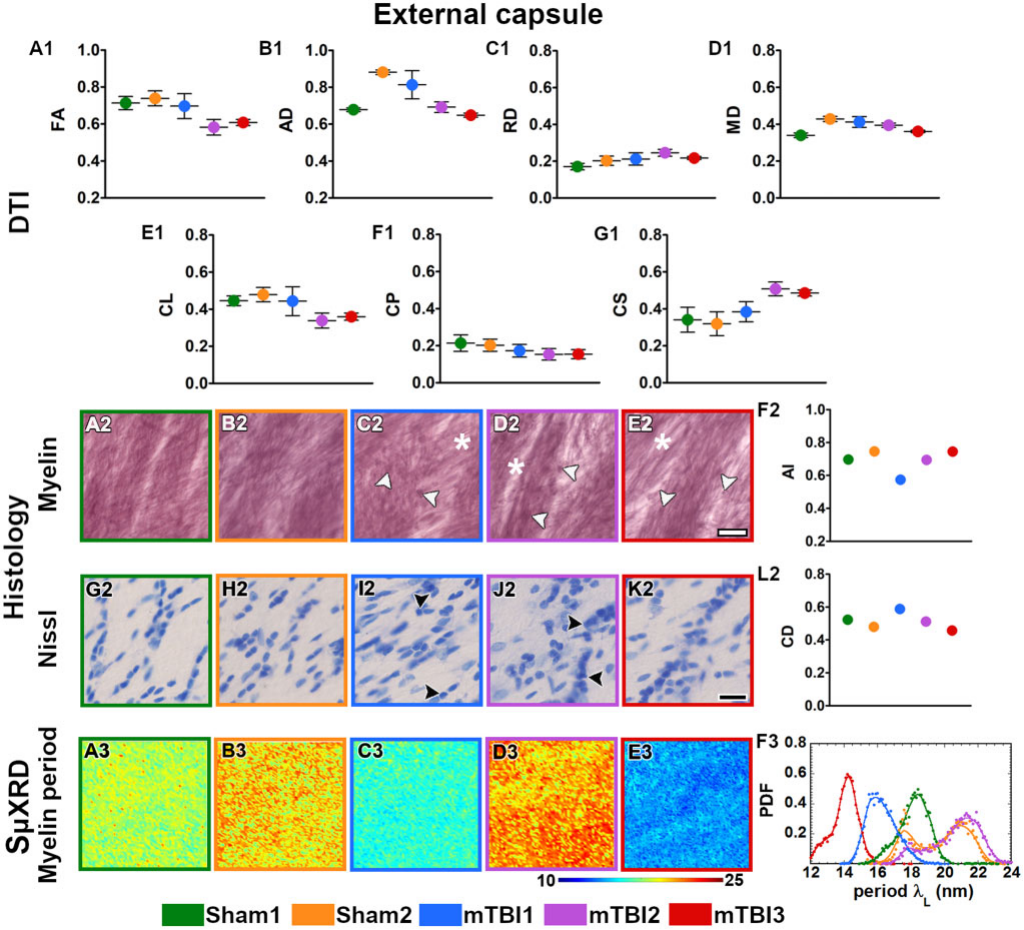
Quantitative analyses in the external capsule. DTI: Ex vivo DTI metrics from the external capsule in sham-operated and mTBI animals **(A1-G1)**. Absolute values AD, RD and MD are scaled (×10^−3^ mm^2^/s). Histology: High-magnification photomicrographs in myelin- **(A2-E2)** and Nissl-stained sections **(G2-K2)** and their corresponding histological derived metrics **(F2, L2)**. Asterisks point to the loss of myelinated axons, white arrowheads to the presence of axonal damage, black arrowheads to the increase in cellularity (gliosis, CD scaled at ×10^−2^ cell/µm^2^). Scale bar: 20 µm. SμXRD: Individual maps of the myelin lamellar period (λ_L_) **(A3-E3)** and probability density function (PDF) of the myelin lamellar period **(F3)** (dots) along the kernel density curves (continuous lines). AD, axial diffusivity; AI, anisotropy index; arb. units, arbitrary units; CD, cell density; CL, linear anisotropy index; CP, planar anisotropy index; CS, spherical anisotropy index; FA, fractional anisotropy; MD, mean diffusivity; RD, radial diffusivity.

**Figure 3.**
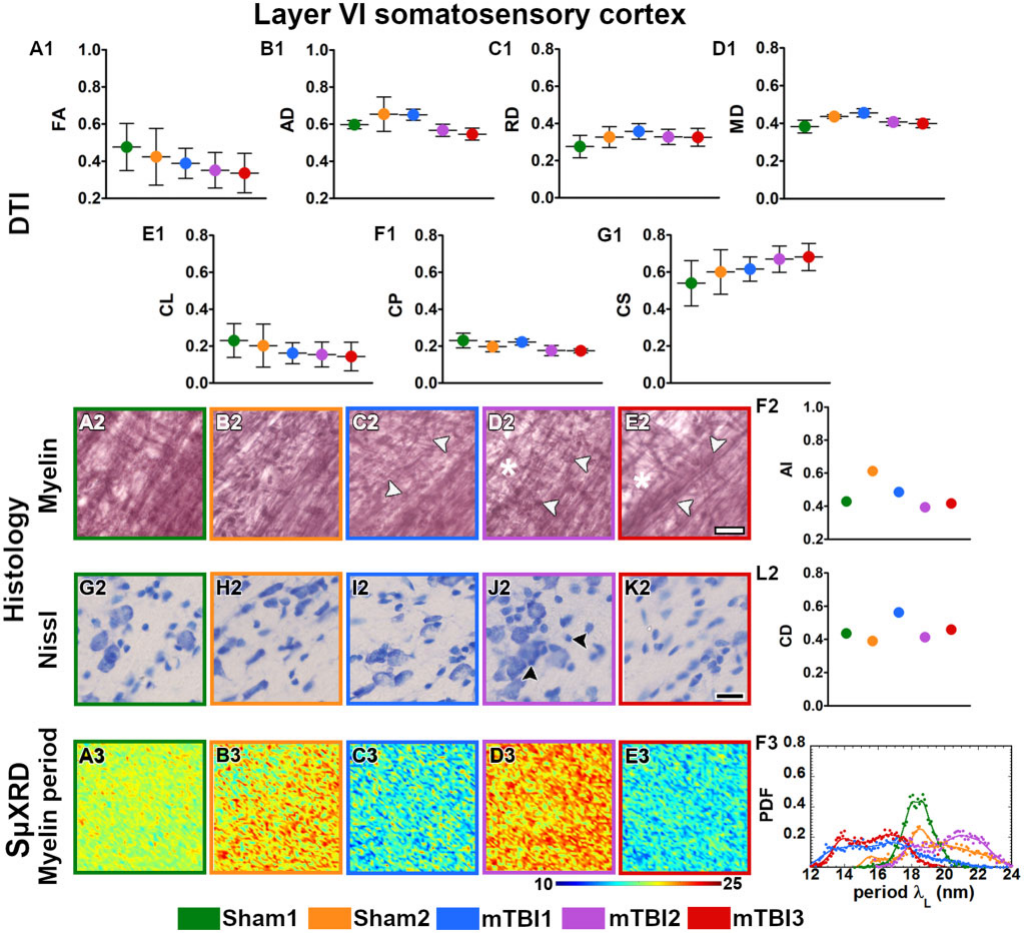
Quantitative analyses in layer VI of the somatosensory cortex. DTI: Ex vivo DTI metrics from the external capsule in sham-operated and mTBI animals **(A1-G1)**. Absolute values AD, RD, and MD are scaled (×10^−3^ mm^2^/s). Histology: High-magnification photomicrographs in myelin- **(A2-E2)** and Nissl-stained sections **(G2-K2)** and their corresponding histological derived metrics **(F2** and **L2)**. Asterisks point to the loss of myelinated axons, white arrowheads to the presence of axonal damage, black arrowheads to the increase in cellularity (gliosis, CD scaled at ×10^−2^ cell/µm^2^). Scale bar: 20 µm. SμXRD: Individual maps of the myelin lamellar period (λ_L_) **(A3-E3)** and probability density function (PDF) of the myelin lamellar period (**F3**) (dots) along the kernel density curves (continuous lines). AD, axial diffusivity; AI, anisotropy index; arb. units, arbitrary units; CD, cell density; CL, linear anisotropy index; CP, planar anisotropy index; CS, spherical anisotropy index; FA, fractional anisotropy; MD, mean diffusivity; RD, radial diffusivity.

**Figure 4.**
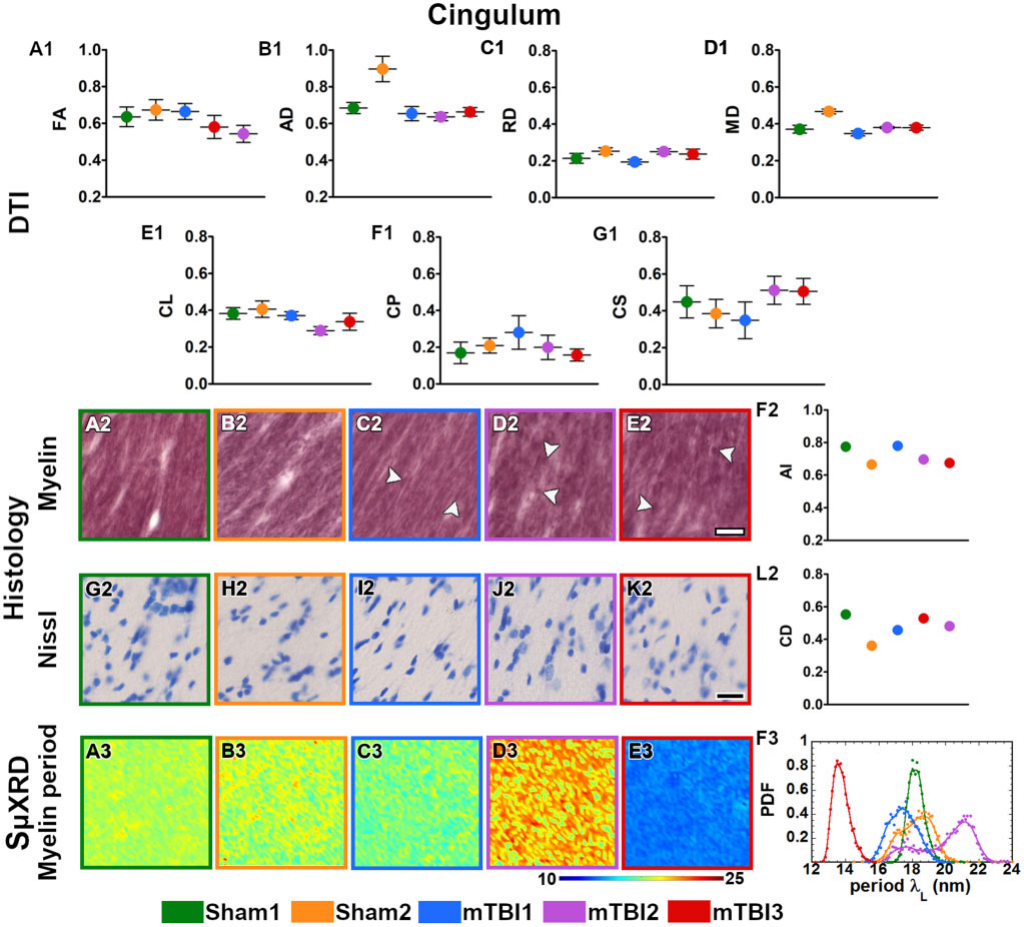
Quantitative analyses in the cingulum. DTI: Ex vivo DTI metrics from the external capsule in sham-operated and mTBI animals (**A1-G1**). Absolute values AD, RD and MD are scaled (×10^−3^ mm^2^/s). Histology: High-magnification photomicrographs in myelin- (**A2-E2**) and Nissl-stained sections (**G2-K2**) and their corresponding histological derived metrics (**F2, L2**). White arrowheads point to the presence of axonal damage. CD scaled at ×10^−2^ cell/µm^2^; scale bar: 20 µm. SμXRD: Individual maps of the myelin lamellar period (λ_L_) (**A3-E3**) and probability density function (PDF) of the myelin lamellar period (**F3**) (dots) along the kernel density curves (continuous lines). AD, axial diffusivity; AI, anisotropy index; arb. units, arbitrary units; CD, cell density; CL, linear anisotropy index; CP, planar anisotropy index; CS, spherical anisotropy index; FA, fractional anisotropy; MD, mean diffusivity; RD, radial diffusivity.

**Figure 5.**
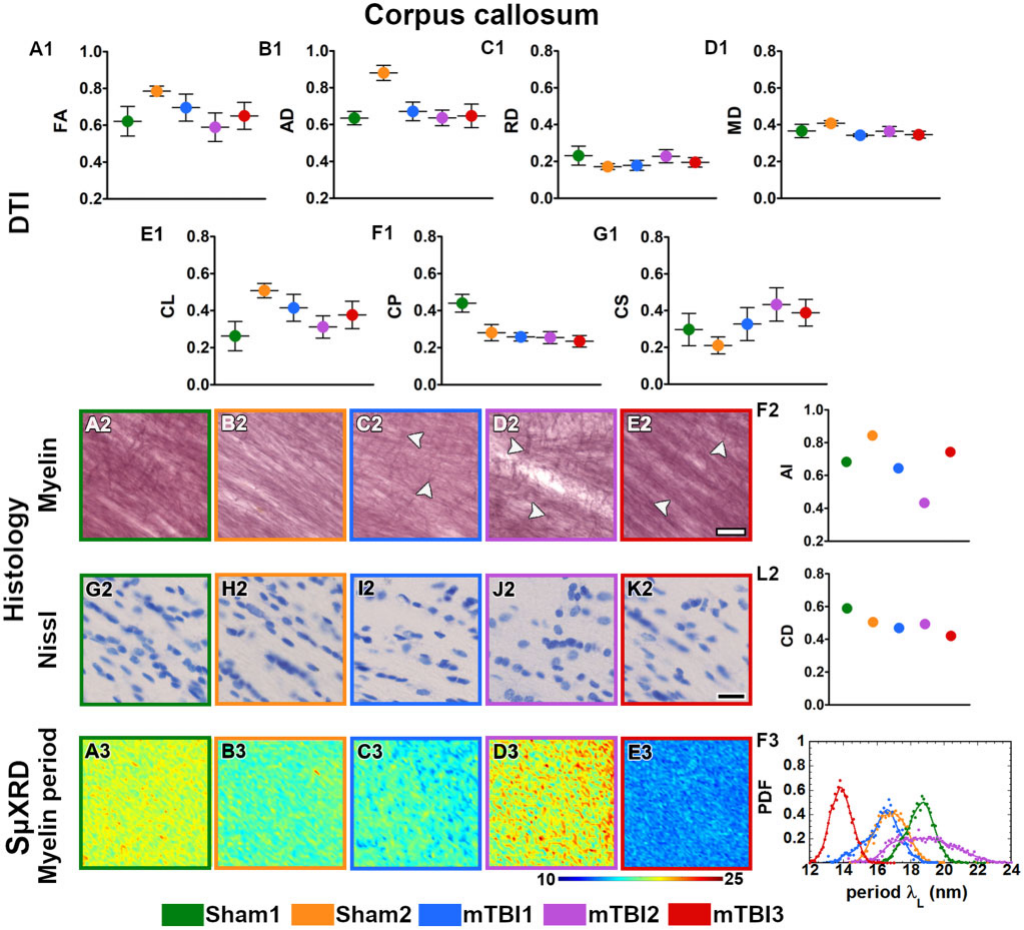
Quantitative analyses in the corpus callosum. DTI: Ex vivo DTI metrics from the external capsule in sham-operated and mTBI animals (**A1-G1**). Absolute values AD, RD, and MD are scaled (×10^−3^ mm^2^/s). Histology: High-magnification photomicrographs in myelin- (**A2-E2**) and Nissl-stained sections (**G2-K2**) and their corresponding histological derived metrics (**F2, L2**). White arrowheads point to the presence of axonal damage. CD scaled at ×10^−2^ cell/µm^2^; scale bar: 20 µm. SμXRD: Individual maps of the myelin lamellar period (λ_L_) (**A3-E3**) and probability density function (PDF) of the myelin lamellar period (**F3**) (dots) along the kernel density curves (continuous lines). AD, axial diffusivity; AI, anisotropy index; arb. units, arbitrary units; CD, cell density; CL, linear anisotropy index; CP, planar anisotropy index; CS, spherical anisotropy index; FA, fractional anisotropy; MD, mean diffusivity; RD, radial diffusivity.

**Figure 6.**
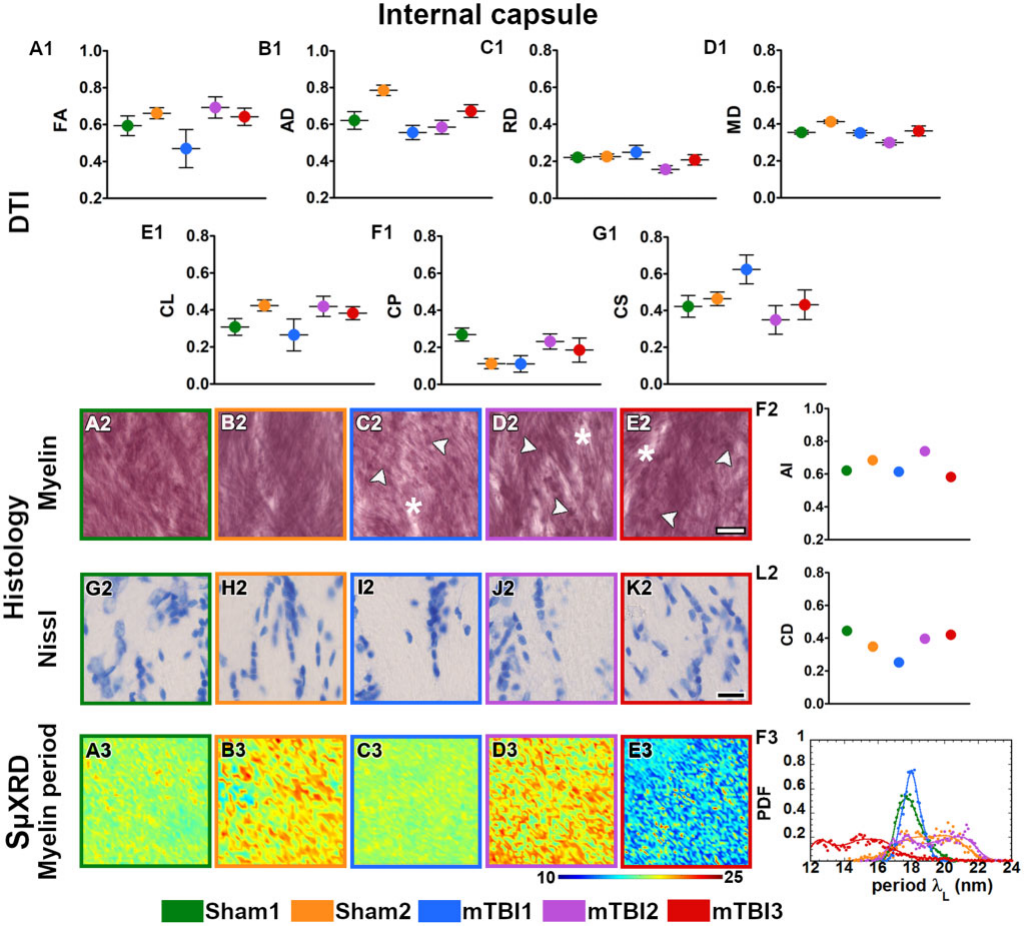
Quantitative analyses in the internal capsule. DTI: Ex vivo DTI metrics from the external capsule in sham-operated and mTBI animals (**A1-G1**). Absolute values AD, RD, and MD are scaled (×10^−3^ mm^2^/s). Histology: High-magnification photomicrographs in myelin- (**A2-E2**) and Nissl-stained sections (**G2-K2**) and their corresponding histological derived metrics (**F2, L2**). Asterisks point to the loss of myelinated axons, white arrowheads to the presence of axonal damage. CD scaled at × 10^−2^ cell/µm^2^; scale bar: 20 µm. SμXRD: Individual maps of the myelin lamellar period (λ_L_) (**A3-E3**) and probability density function (PDF) of the myelin lamellar period (**F3**) (dots) along the kernel density curves (continuous lines). AD, axial diffusivity; AI, anisotropy index; arb. units, arbitrary units; CD, cell density; CL, linear anisotropy index; CP, planar anisotropy index; CS, spherical anisotropy index; FA, fractional anisotropy; MD, mean diffusivity; RD, radial diffusivity.

All the mTBI animals appeared to have lower values in FA, CL and CP parameters (Fig. 2A1, E1, and F1) whereas they displayed higher RD and CS values in the external capsule (Fig. 2C1, G1) as compared to sham-operated ones. In layer VI of the somatosensory cortex, FA and CL values appeared to be lower (Fig. 3A1, E1), but higher in CS (Fig. 3G1) in mTBI animals. Moreover, the mTBI animals appeared to have lower AD and CL values in the cingulum (Fig. 4B1, E1), whereas they exhibited lower CP and higher CS values in the corpus callosum (Fig. 5F1, G1). In the internal capsule, the values of DTI parameters from the mTBI animals did not differ from their sham-operated counterparts (Fig. 6A1–G1).

### Evidence of axonal damage, loss of myelinated axons and gliosis after mTBI shown by histological analyses of myelin- and Nissl-stained sections

Axonal damage, seen as darker spots in myelin staining, was present in all the brain areas of the mTBI animals analyzed in this study (Figs. 2–6C2–E2). mTBI rats appeared to have lower AI values in the external capsule (mTBI1 and 2; Fig. 2F2), layer VI of the somatosensory cortex (mTBI2 and 3; Fig. 3F2), corpus callosum (mTBI1 and 2; Fig. 5F2) and internal capsule (mTBI1 and 3; Fig. 6F2) as compared to sham-operated animals. Additionally, we observed a reduced density of myelin staining related to a loss of myelinated axons in the external capsule (Fig. 2C2–E2), layer VI of the somatosensory cortex (Fig. 3D2, 3E2) and internal capsule (Fig. 6C2–E2). In Nissl staining, a visual examination revealed an increase in cellularity or gliosis in the external capsule (Fig. 2I2, J2) and layer VI of the somatosensory cortex (Fig. 3J2). CD values appeared higher in the external capsule (mTBI1; Fig. 2L2) and layer VI of the somatosensory cortex (mTBI1 and 3; Fig. 3L2).

### Alterations in the ultrastructure of the myelin by changes in SµXRD metrics after mTBI

**Figure 7.**
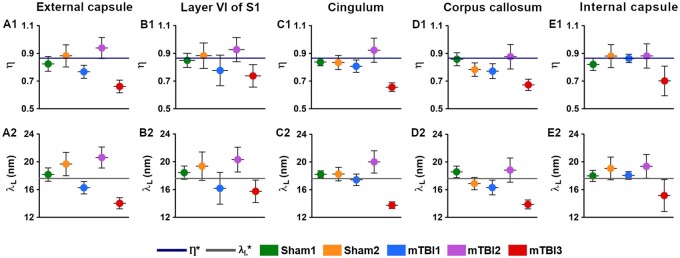
Commensurability (η) and lamellar period (λ_L_) values from the external capsule **(A1, A2)**, layer VI of the somatosensory cortex **(B1, B2)**, cingulum **(C1, C2)**, corpus callosum **(D1, D2)**, and internal capsule **(E1, E2).** Based on a literature review, the thick dark blue line represents the value for the normal state of myelin (η* = 0.866; [48]), and the thick grey line for the normal lamellar period value (λ_L_* = 17.6 nm; [47]). S1, primary somatosensory cortex.

All animals examined in this study appeared to have the predominant characteristic multilamellar structural phase of myelin with a minority exhibiting a dispersed hexagonal phase (Fig. 1). Here, we report the results obtained from the predominant multilamellar phase of the myelin. According to the literature, we considered standard values for the lamellar period and commensurability as λ_L_* = 17.6 nm (47) and η* = 0.866 (48), respectively. Sham1 showed the values closest to the standard lamellar period (Fig. 7A2–E2; Supplementary Table S1) and commensurability of myelin (Fig. 7A1–E1; Supplementary Table S1) throughout all the brain areas. After mTBI, we found compressed λ_L_ < λ_L_* (mTBI3) and expanded λ_L_ > λ_L_* (mTBI2) lamellar periods in all the brain areas (Fig. 7A2–E2; Supplementary Table S1). On the other hand, we also found fluctuating λ_L_ values around λ_L_* after the sham-operation and the mTBI although this tended to vary between the different brain areas studied (Sham2 and mTBI1; Fig. 7A2–7E2; Supplementary Table S1). Based on the standard commensurability η*, mTBI animals appeared to have lower η values in all the examined brain areas (mTBI1 and 3; Fig. 7A1–E1; Supplementary Table S1). Furthermore, after the sham-operation and mTBI, we also found η values close to η* (Sham2 and mTBI2; Fig. 7A1–E1; Supplementary Table S1). In this regard, Sham2 showed lower η values in the corpus callosum, and mTBI2 exhibited SD values larger than, for example, than those of Sham1, which might reflect extensive heterogeneity in the form of myelin damage. In summary, mTBI animals showed larger deviations of η and λ_L_ values from η* and λ_L_* than sham-operated animals. It seems likely that the profiles obtained in the 3 mTBI animals might reflect different responses to the brain injury, as well as different ongoing degenerative and repair processes at this experimental time point.

### Relationship between DTI, histological and SμXRD imaging modalities

We used multiple linear regression to assess if DTI could explain changes in the histological parameters estimated from Nissl and myelin staining. DTI showed a correlation with AI and explained over 55% of the variation in this parameter (*R*^2^ = 0.566 *R*^2^ adj = 0.452, 95% CI [0.100–0.670]), whereas DTI explained over 25% in CD (*R*^2^ = 0.297, *R*^2^ adj = 0.113, 95% CI [0.000–0.440]) (Table 1). We then evaluated how well DTI could explain diffraction parameters, including the content and the period (C_L_, C_H_, λ_L_, and λ_H_). In this regard, DTI explained over 25% of the variation in C_L_ (*R*^2^ = 0.268, *R*^2^ adj = 0.076, 95% CI [0.000–0.410]) and C_H_ (*R*^2^ = 0.267, *R*^2^ adj = 0.074, 95% CI [0.000–0.410]) (Table 2). In the lamellar and hexagonal phase periods, DTI explained only over 4% of the variation in λ_L_ (*R*^2^ = 0.042, *R*^2^ adj = −0.211, 95% CI [0.000–0.070]) and λ_H_ (*R*^2^ = 0.073, *R*^2^ adj = −0.171, 95% CI [0.000–0.120]) (Table 2). Histology could only explain some of the diffraction parameters (over 20% of the variation in C_L_ (*R*^2^ = 0.274, *R*^2^ adj = 0.208, 95% CI [0.000–0.480]) and C_H_ (*R*^2^ = 0.233, *R*^2^ adj = 0.163, 95% CI [0.000–0.450]) (Table 3). In the lamellar and hexagonal phase periods, histology explained only over 1% in λ_L_ (*R*^2^ = 0.027, *R*^2^ adj = −0.062, 95% CI [0.000–0.190]) and λ_H_ (*R*^2^ = 0.012, *R*^2^ adj = −0.078, 95% CI [0.000–0.120]) (Table 3). Additionally, we assessed whether histology could explain DTI parameters and found that histology explained over 50% of their variation in FA and CS, whereas it accounted for only over 6% of the variation in AD, MD and CP (Supplementary Table S2). Subsequently, we investigated whether diffraction could explain histological and DTI parameters. With respect to the histological parameters, diffraction only explained over 30% of the variation in AI and 4% in CD (Supplementary Table S3). With respect to the DTI parameters, diffraction explained over 20% of the variation in FA, over 14% in AD and CS, and over 4% in MD and CP (Supplementary Table S4). These results suggest that DTI and histology methodologies could explain each other to some extent, whereas diffraction did not reflect either the histology findings or the DTI. These results suggest that DTI and histology have a limited ability to assess the nanoscale alterations shown by SµXRD. On the other hand, the assessment of the pathophysiology of mTBI and thus also the interpretation of DTI can be improved by applying several imaging modalities.

**Table 1.** Multiple linear regression analysis of the relationship between DTI and histological parameters

	*R* ^2^ (95% CI)	*R* ^2^ adj	*F*	*t* (FA)	*t* (AD)	*t* (MD)	*t* (CP)	*t* (CS)
	p			p	p	p	p	p
AI	0.566 (0.100–0.670)**	0.452	4.965	0.885	−0.181	0.159	0.049	0.169
	0.004			0.387	0.859	0.875	0.961	0.868
CD	0.297 (0.000–0.440)	0.113	1.609	0.875	−1.496	1.586	−0.564	−0.730
	0.206			0.392	0.151	0.129	0.579	0.474

p values for multiple linear regression tests between DTI (predictors) and histological (dependent variable) parameters. Significant value is highlighted with asterisks (**p < 0.01; multiple linear regression model). RD and CL were excluded variables based on the linear regression model; data not shown in this table.

AD, axial diffusivity; AI, anisotropy index; CD, cell density; CP, planar anisotropy index; CS, spherical anisotropy index; FA, fractional anisotropy; MD, mean diffusivity.

**Table 2.** Multiple linear regression analysis of the relationship between DTI and diffraction parameters

	*R* ^2^ (95% CI)	*R* ^2^ adj	*F*	*t* (FA)	*t* (AD)	*t* (MD)	*t* (CP)	*t* (CS)
	p			p	p	p	p	p
C_L_	0.268 (0.000–0.410)	0.076	1.393	1.597	0.492	−0.408	1.250	1.501
	0.271			0.127	0.628	0.688	0.226	0.150
λ_L_	0.042 (0.000–0.070)	−0.211	0.165	−0.016	0.362	−0.296	0.410	0.333
	0.972			0.987	0.721	0.770	0.686	0.743
C_H_	0.267 (0.000–0.410)	0.074	1.385	1.791	0.352	−0.279	1.311	1.527
	0.274			0.089	0.729	0.784	0.206	0.143
λ_H_	0.073 (0.000–0.120)	−0.171	0.300	−0.195	0.184	−0.115	0.051	−0.047
	0.907			0.847	0.856	0.910	0.960	0.963

p values for multiple linear regression tests between DTI (predictors) and diffraction parameters (dependent variables). RD and CL were excluded variables based on the linear regression model; data not shown in this table.

AD, axial diffusivity; CP, planar anisotropy index; CS, spherical anisotropy index; FA, fractional anisotropy; C_H_, hexagonal phase content; λ_H_, hexagonal phase period; C_L_, lamellar phase content; λ_L_, lamellar phase period; MD, mean diffusivity.

**Table 3.** Multiple linear regression analysis of the relationship between histological and diffraction parameters

	*R* ^2^ (95% CI)	*R* ^2^ adj	*F*	*t* (AI)	*t* (CD)
	p			p	p
C_L_	0.274 (0.000–0.480)*	0.208	4.150	2.862**	0.128
	0.030			0.009	0.899
λ_L_	0.027 (0.000–0.190)	−0.062	0.302	−0.302	−0.693
	0.742			0.765	0.496
C_H_	0.233 (0.000–0.450)	0.163	3.343	2.582*	−0.034
	0.054			0.017	0.973
λ_H_	0.012 (0.000–0.120)	−0.078	0.136	0.519	−0.080
	0.874			0.609	0.937

p values for multiple linear regression tests between histological (predictors) and diffraction (dependent variables) parameters are highlighted with asterisks (*p < 0.05; **p < 0.01; multiple linear regression model).

AI, anisotropy index; CD, cell density; C_H_, hexagonal phase content; λ_H_, hexagonal phase period; C_L_, lamellar phase content; λ_L_, lamellar phase period.

## DISCUSSION

In this study, we evaluated the potential of DTI to detect tissue damage after an experimental mTBI in combination with a multiscale tissue assessment by histology and SμXRD. We found that DTI may reflect changes in white and grey matter areas after the injury; our histological analyses of myelin and Nissl staining illustrated axonal damage, loss of myelinated axons and gliosis. In mTBI animals, SμXRD also demonstrated alterations in the local structure of myelin, such as periodicity and content. In summary, these findings demonstrate that DTI distinguished the microstructural changes associated with loss of myelinated axons and gliosis only to a limited extent. Furthermore, the axonal injury and the damage of myelin visible by light microscopy and by SµXRD were not detected by DTI.

In the present study, DTI showed moderate alterations in the values in mTBI animals as compared to sham-operated ones in the areas closest to the primary injury, that is, the external capsule and somatosensory cortex. DTI parameters from the corpus callosum and cingulum in mTBI animals exhibited moderate deviations from the DTI values in sham-operated animals, while parameters from the internal capsule did not show any apparent changes in DTI values between these animal groups. The animals selected for these analyses were representative of larger groups included in our previous studies (32, 33). When utilizing in vivo DTI at 3 days after the injury, we found significant differences in DTI parameters in the external capsule, somatosensory cortex, corpus callosum, and cingulum, but not in the internal capsule (32). On day 28, in vivo and on day 35 ex vivo, the differences between sham-operated and mTBI animals were still evident in the external capsule, corpus callosum and cingulum, but no differences could be detected in the somatosensory cortex or the internal capsule (32, 33). Similarly, other investigators have reported significant differences in the corpus callosum, cingulum, external capsule, and the somatosensory cortex in the acute phase of mTBI in vivo (25, 51–54) as well as differences in the corpus callosum and cingulum in the subacute phase both in vivo (29–31) and ex vivo (27).

DTI changes have been associated with axonal damage in white matter (27, 32, 33) and with astro- and microgliosis in white and grey matter after mTBI (30, 31, 51, 55). The correlation between DTI and the tissue parameters extracted from advanced histological analyses, such as ST, automated cell counting, or morphological skeleton-based approaches, can improve the characterization of DTI in terms of tissue microstructure (31–33, 49). The results of the present study suggest that DTI may reflect tissue changes occurring at the cellular level after mTBI such as loss of myelinated axons and gliosis, but it is less successful at detecting signs of an axonal injury at the subcellular level.

SμXRD distinguished alterations in the ultrastructure of myelin, which may be an indication of either ongoing degenerating or regenerating processes in the myelin sheath of axons in mTBI brains. SμXRD is a technique which can sensitively detect local fluctuations in the dynamics of the ultrastructure of myelin in the central and peripheral nervous system (56–58). Our data showed the characteristic multilamellar phase of myelin in all the sham-operated and mTBI animals as described in the literature (59, 60). After mTBI, we observed fluctuations in the lamellar period associated with expanded and compressed phases of the myelin structure. Previous studies have also reported variations in the myelin structural phases in response to changes in harsh environmental conditions such as temperature, rehydration, chemical treatment, etc. (61–63). Furthermore, changes were also evident in commensurability in mTBI animals related to an altered membrane packing of myelin. Other investigators have reported transitions from the lamellar to the inverted hexagonal phase in multiple sclerosis (64, 65), as well as the presence of an hexatic phase in Parkinson disease (66), which also are indicative of a structural instability in myelin due to pathology.

When comparing DTI and SμXRD outcomes, we found that DTI did not explain well changes in the ultrastructure of myelin detected by SμXRD. Previously, Georgiadis et al (67) evaluated the relationship between dMRI and 3D SAXS in healthy tissue. These authors found strong correlations when comparing fiber orientation distributions and anisotropy of myelinated axons at the microstructural level using 3D and the average q-range orientation derived from dMRI; instead, the present study focused on changes in the ultrastructural organization after mTBI by using 2D SμXRD, showing limited sensitivity of DTI to detect ultrastructural myelin changes.

We conducted extensive assessment of the tissue damage at the micro- and nanoscale levels, but a few limitations should be taken into consideration. The main limitation of our study is the sample size (sham = 2; mTBI = 3), which revealed variability between animals in DTI, histology and SµXRD findings indicating uncertainty of the statistical parameters. Second, it is important to consider that chemical fixation can potentially affect the tissue microstructural properties and therefore, in vivo and ex vivo DTI results should be compared (68, 69). However, it is worth mentioning that the use of ex vivo DTI acquisitions allows higher resolution as well as no susceptibility to motion artifacts, thereby providing additional information about the tissue properties that can be translated to in vivo settings (70). Third, in this study, we used a single tensor model of diffusion that might underestimate the complex pathological tissue alterations that provides a better estimation about single fiber population within a voxel, but it is more limited to differentiate complex cellular features in regions with crossing fibers (71). Because DTI averages out signal in a voxel, this technique is limited to reflect ultrastructural properties of the myelin as shown by SµXRD. The implementation of more advanced dMRI approaches might improve the characterization of these microstructural alterations in white and grey matter (72, 73), and the comparisons between dMRI, histological and diffraction parameters. Furthermore, if we had been able to exploit 3D light microscopy and quantitative histological analyses, this could have provided a more complete 3D perspective of the DTI data (74, 75). Regarding the limitations of SµXRD, the implementation of 3D SAXS acquisitions (67, 76, 77) might enhance the comparisons in future studies in terms of the tissue’s orientation properties by reconstructing the orientation of an average q-range, although this might not be sensitive at detecting ultrastructural alterations. Finally, it is challenging to make comparisons among these imaging modalities because the tissue parameters are extracted at different length scales and resolutions, although they do offer complementary information of the micro- and ultrastructure of the tissue ex vivo.

In conclusion, the findings suggest that DTI was moderately sensitive at distinguishing changes at the microstructural level in the subacute phase of mTBI, such as gliosis and loss of myelinated axons, but was less successful at detecting features at the nanometer scale such as axonal damage and ultrastructural-level alterations in myelin. The adoption of a multimodal imaging approach can offer new ways to study tissue changes after mild brain injury and may allow a better interpretation of DTI findings in future mTBI studies.

## FUNDING

This work was supported by the Academy of Finland (#323385 [A.S.], #316258 [J.T.]), and Vilho, Yrjö and Kalle Väisälä Foundation of the Finnish Academy of Science and Letters.

## Supplementary Material

nlac100_Supplementary_Data
