# Supplementary material for: A multiscale tissue assessment in a rat model of mild traumatic brain injury
Source: J Neuropathol Exp Neurol. 2022 Nov 4;82(1):71–83. doi: 10.1093/jnen/nlac100 (PMC9764078; doi:10.1093/jnen/nlac100)
Supplement: nlac100_Supplementary_Data [file nlac100_supplementary_data.docx]

**
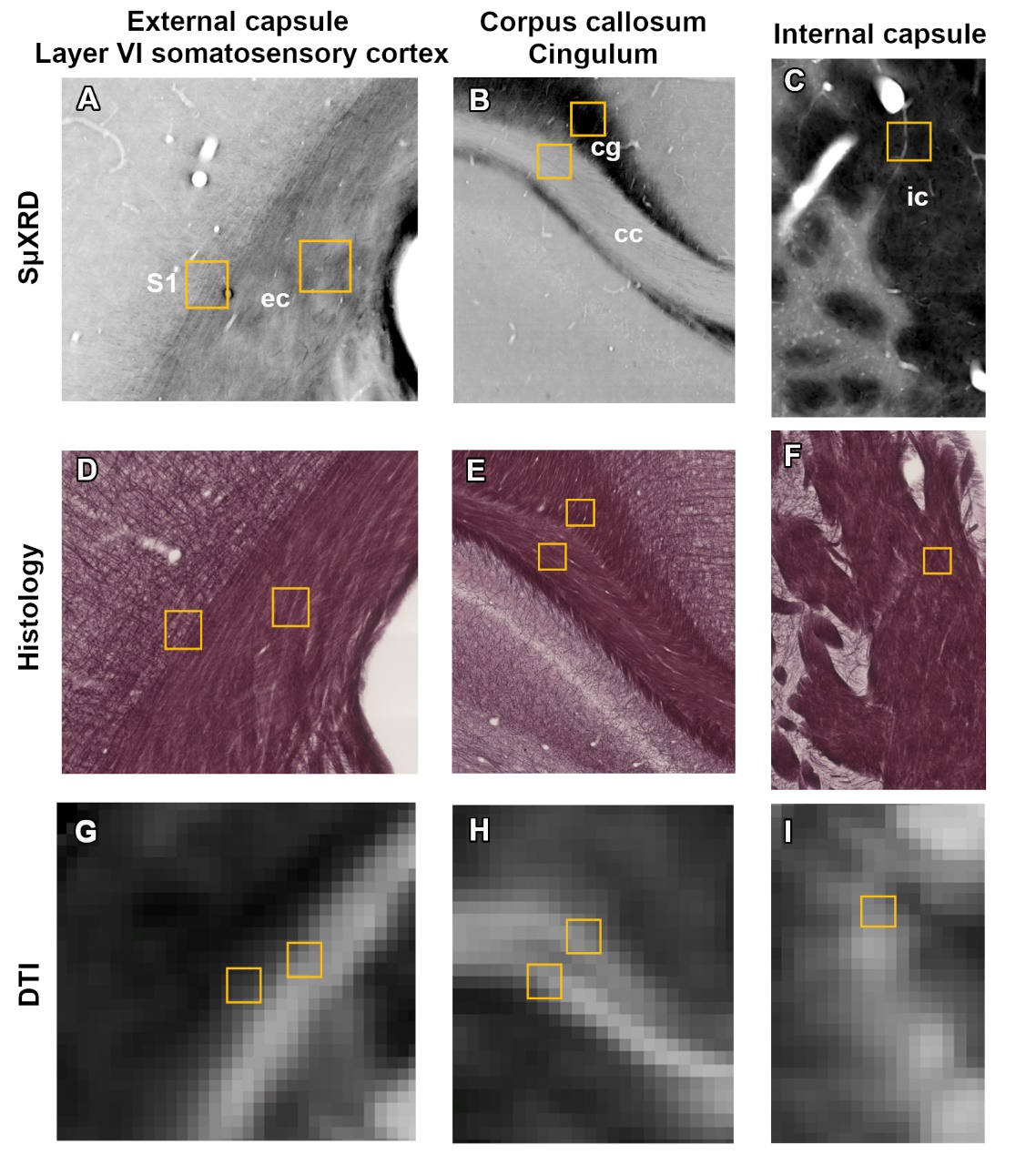
Supplementary Figure 1.** Representative photomicrographs from a sham animal showing the ROIs included for DTI, histological, and SμXRD analyses at approximately -2.00 mm from bregma in the ipsilateral side of the brain. In the SμXRD transmission mean images the ROI area selected for analysis was 74 × 64 pixels in the layer VI of somatosensory cortex (**A**), 99 × 82 pixels in the external capsule (**A**), 50 × 50 pixels in the corpus callosum and cingulum (**B**), and 50 × 50 pixels in the internal capsule (**C**). In the histological photomicrographs, the ROI area was 814 × 824 pixels in layer VI of the somatosensory cortex (**D**), 832 × 849 pixels in the external capsule (**D**), 800 × 800 pixels in the corpus callosum and cingulum (**E**), and 800 × 800 pixels in the internal capsule (**F**). In the DTI data, we outlined 8 pixels in layer VI of the somatosensory cortex (**G**), 7 pixels in the external capsule (**G**), 6 pixels in the corpus callosum and cingulum (**H**) and in the internal capsule (**I**). Abbreviations: cc, corpus callosum; cg, cingulum; DTI, diffusion tensor imaging; ec, external capsule; ic, internal capsule; S1, primary somatosensory cortex; SμXRD, Scanning micro-X-Ray diffraction.

**Supplementary Table 1. Myelin content and period for lamellar and hexagonal phases, and commensurability values for sham-operated and mTBI animals in all brain regions**

|  | **C_L_ ± SD (arb.units)** | **λ_L_ ± SD (nm)** | **C_H_ ± SD (arb.units)** | **λ_H_ ± SD (nm)** | **η ± SD** | **η - η*** |
| --- | --- | --- | --- | --- | --- | --- |
| **All brain areas** | | | | | | |
| **Sham1** | 75.750 ± 41.279 | 18.279 ± 0.233 | 23.017 ± 10.063 | 21.832 ± 0.162 | 0.838 ± 0.016 | -0.028 |
| **Sham2** | 81.307 ± 51.970 | 18.649 ± 1.118 | 21.395 ± 12.337 | 21.883 ± 0.289 | 0.852 ± 0.044 | -0.013 |
| **mTBI1** | 46.071 ± 43.564 | 16.851 ± 0.842 | 16.031 ± 15.236 | 21.144 ± 0.303 | 0.797 ± 0.040 | -0.069 |
| **mTBI2** | 85.635 ± 35.041 | 19.827 ± 0.728 | 20.151 ± 9.304 | 21.830 ± 0.205 | 0.909 ± 0.028 | 0.043 |
| **mTBI3** | 60.293 ± 49.345 | 14.508 ± 0.889 | 11.967 ± 11.254 | 21.175 ± 0.376 | 0.685 ± 0.034 | -0.180 |
| **External capsule** | | | | | | |
| **Sham1** | 76.221 ± 24.999 | 18.170 ± 0.966 | 19.033 ± 10.121 | 22.069 ± 0.530 | 0.824 ± 0.053 | -0.042 |
| **Sham2** | 81.759 ± 29.468 | 19.676 ± 1.675 | 18.237 ± 11.354 | 22.322 ± 0.393 | 0.882 ± 0.080 | 0.016 |
| **mTBI1** | 46.698 ± 18.809 | 16.271 ± 0.890 | 19.867 ± 9.881 | 21.220 ± 0.371 | 0.767 ± 0.047 | -0.099 |
| **mTBI2** | 99.199 ± 38.096 | 20.625 ± 1.513 | 19.210 ± 13.187 | 21.988 ± 0.422 | 0.939 ± 0.076 | 0.073 |
| **mTBI3** | 85.083 ± 34.370 | 14.026 ± 0.804 | 20.276 ± 15.444 | 21.248 ± 0.592 | 0.661 ± 0.046 | -0.205 |
| **Layer VI of somatosensory cortex** | | | | | | |
| **Sham1** | 34.350 ± 17.836 | 18.440 ± 0.950 | 13.182 ± 7.846 | 21.728 ± 0.443 | 0.849 ± 0.051 | -0.017 |
| **Sham2** | 35.102 ± 17.199 | 19.372 ± 2.034 | 9.621 ± 5.588 | 21.938 ± 0.462 | 0.883 ± 0.092 | 0.017 |
| **mTBI1** | 12.259 ± 29.103 | 16.185 ± 2.290 | 6.120 ± 14.106 | 20.848 ± 0.382 | 0.776 ± 0.110 | -0.089 |
| **mTBI2** | 55.288 ± 20.467 | 20.312 ± 1.782 | 11.496 ± 7.953 | 21.940 ± 0.420 | 0.926 ± 0.087 | 0.060 |
| **mTBI3** | 28.190 ± 30.534 | 15.750 ± 1.620 | 5.789 ± 21.804 | 21.389 ± 0.627 | 0.737 ± 0.081 | -0.129 |
| **Corpus callosum** | | | | | | |
| **Sham1** | 50.152 ± 16.509 | 18.573 ± 0.821 | 16.413 ± 8.933 | 21.667 ± 0.438 | 0.858 ± 0.047 | -0.008 |
| **Sham2** | 54.427 ± 16.956 | 16.886 ± 0.890 | 12.605 ± 6.842 | 21.588 ± 0.526 | 0.783 ± 0.049 | -0.083 |
| **mTBI1** | 22.925 ± 13.442 | 16.316 ± 1.063 | 3.327 ± 4.271 | 21.150 ± 0.377 | 0.772 ± 0.054 | -0.094 |
| **mTBI2** | 41.838 ± 46.080 | 18.834 ± 1.749 | 11.059 ± 22.907 | 21.526 ± 0.559 | 0.876 ± 0.089 | 0.010 |
| **mTBI3** | 38.459 ± 13.214 | 13.868 ± 0.640 | 5.349 ± 5.980 | 20.641 ± 0.491 | 0.673 ± 0.041 | -0.193 |
| **Cingulum** | | | | | | |
| **Sham1** | 142.431 ± 33.511 | 18.234 ± 0.499 | 37.716 ± 14.821 | 21.778 ± 0.276 | 0.837 ± 0.027 | -0.028 |
| **Sham2** | 169.142 ± 57.330 | 18.259 ± 0.977 | 40.382 ± 23.608 | 21.908 ± 0.394 | 0.834 ± 0.051 | -0.032 |
| **mTBI1** | 120.742 ± 42.011 | 17.436 ± 0.820 | 40.879 ± 19.821 | 21.607 ± 0.397 | 0.807 ± 0.044 | -0.059 |
| **mTBI2** | 121.096 ± 39.264 | 20.011 ± 1.604 | 26.930 ± 18.004 | 21.711 ± 0.567 | 0.923 ± 0.087 | 0.057 |
| **mTBI3** | 134.838 ± 30.769 | 13.753 ± 0.492 | 27.354 ± 15.706 | 20.983 ± 0.403 | 0.656 ± 0.031 | -0.210 |
| **Internal capsule** | | | | | | |
| **Sham1** | 75.595 ± 25.810 | 17.979 ± 0.801 | 28.741 ± 12.262 | 21.919 ± 0.327 | 0.821 ± 0.043 | -0.045 |
| **Sham2** | 66.106 ± 130.030 | 19.053 ± 1.633 | 26.129 ± 62.764 | 21.659 ± 0.551 | 0.881 ± 0.083 | 0.015 |
| **mTBI1** | 27.732 ± 8.106 | 18.046 ± 0.559 | 9.961 ± 2.761 | 20.898 ± 0.194 | 0.864 ± 0.030 | -0.002 |
| **mTBI2** | 110.755 ± 44.535 | 19.350 ± 1.700 | 32.062 ± 18.778 | 21.985 ± 0.473 | 0.881 ± 0.087 | 0.015 |
| **mTBI3** | 14.895 ± 35.747 | 15.146 ± 2.300 | 1.067 ± 14.349 | 21.613 ± 0.646 | 0.701 ± 0.107 | -0.165 |

According to the literature, we considered that the standard value for the normal state of the myelin structure is η^*^= 0.866 (48). Abbreviations: arb. units, arbitrary units; C_H_, hexagonal phase content; λ_H_, hexagonal phase period; C_L_, lamellar phase content; λ_L_, lamellar phase period; SD, standard deviation.

**Supplementary Table 2. Multiple linear regression analysis of the relationship between histological and DTI parameters**

|  | **R^2^ (95% CI)**  ***p*** | **R^2^ adj** | **F** | **t (AI)**  ***p*** | **t (CD)**  ***p*** |
| --- | --- | --- | --- | --- | --- |
| **FA** | 0.574 (0.230, 0.710)***  8.500×10^-5^ | 0.535 | 14.803 | 5.271***  2.70×10^-5^ | 0.976  0.340 |
| **AD** | 0.226 (0.000, 0.440)  0.060 | 0.155 | 3.204 | 2.389*  0.026 | 0.669  0.511 |
| **MD** | 0.065 (0.000, 0.260)  0.475 | -0.019 | 0.771 | -1.206  0.241 | 0.380  0.708 |
| **CP** | 0.178 (0.000, 0.400)  0.115 | 0.104 | 2.386 | 0.468  0.644 | 2.096*  0.048 |
| **CS** | 0.516 (0.160, 0.670)***  3.450×10^-4^ | 0.471 | 11.705 | -4.392***  2.32×10^-4^ | -1.717  0.100 |

Statistically *p* values for multiple linear regression tests between histological (predictors) and DTI (dependent variables) parameters are highlighted with asterisks (**p* < 0.05; ****p* < 0.001; multiple linear regression model). RD and CL were not included in the regression model, since according to the previous linear regression models, these should be excluded based on collinearity. Abbreviations: AD, axial diffusivity; AI, anisotropy index; CD, cell density; CP, planar anisotropy index; CS, spherical anisotropy index; FA, fractional anisotropy; MD, mean diffusivity.

**Supplementary Table 3. Multiple linear regression analysis of the relationship between diffraction and histological parameters**

|  | **R^2^ (95% CI)**  ***p*** | **R^2^ adj** | **F** | **t (C_L_)**  ***p*** | **t (λ_L_)**  ***p*** | **t (C_H_)**  ***p*** | **t (λ_H_)**  ***p*** |
| --- | --- | --- | --- | --- | --- | --- | --- |
| **AI** | 0.314 (0.000, 0.480)  0.096 | 0.177 | 2.287 | 0.912  0.373 | -0.957  0.350 | 0.294  0.771 | 0.364  0.720 |
| **CD** | 0.050 (0.000, 0.120)  0.899 | -0.140 | 0.262 | 0.207  0.838 | -0.931  0.363 | -0.138  0.892 | 0.606  0.552 |

Statistically *p* values for multiple linear regression tests between diffraction (predictors) and histological (dependent variables) parameters are shown (multiple linear regression model). Abbreviations: AI, anisotropy index; CD, cell density; C_H_, hexagonal phase content; λ_H_, hexagonal phase period; C_L_, lamellar phase content; λ_L_, lamellar phase period.

**Supplementary Table 4. Multiple linear regression analysis of the relationship between diffraction and DTI parameters**

|  | **R^2^ (95% CI)**  ***p*** | **R^2^ adj** | **F** | **t (C_L_)**  ***p*** | **t (λ_L_)**  ***p*** | **t (C_H_)**  ***p*** | **t (λ_H_)**  ***p*** |
| --- | --- | --- | --- | --- | --- | --- | --- |
| **FA** | 0.235 (0.000, 0.400)  0.229 | 0.082 | 1.539 | -0.094  0.926 | -1.646  0.115 | 0.750  0.462 | 1.378  0.183 |
| **AD** | 0.144 (0.000, 0.300)  0.514 | -0.027 | 0.843 | 0.444  0.662 | -0.890  0.384 | -0.020  0.984 | 1.129  0.272 |
| **MD** | 0.044 (0.000, 0.100)  0.919 | -0.147 | 0.230 | 0.519  0.610 | 0.568  0.576 | -0.640  0.529 | -0.018  0.986 |
| **CP** | 0.070 (0.000, 0.170)  0.822 | -0.116 | 0.377 | -1.055  0.304 | -0.434  0.669 | 0.814  0.425 | 0.746  0.464 |
| **CS** | 0.187 (0.000, 0.350)  0.363 | 0.024 | 1.147 | 0.471  0.643 | 1.568  0.133 | -0.851  0.405 | -1.519  0.144 |

Statistically *p* values for multiple linear regression tests between diffraction (predictors) and DTI (dependent variables) parameters are highlighted with asterisks (**p* < 0.05; multiple linear regression model). RD and CL were not included in the regression model, since according to the previous linear regression models, they should be excluded based on collinearity. Abbreviations: AD, axial diffusivity; CP, planar anisotropy index; CS, spherical anisotropy index; FA, fractional anisotropy; C_H_, hexagonal phase content; λ_H_, hexagonal phase period; C_L_, lamellar phase content; λ_L_, lamellar phase period; MD, mean diffusivity.
